# The influence of tissue pH and RNA integrity number on gene expression of human postmortem brain

**DOI:** 10.3389/fpsyt.2023.1156524

**Published:** 2023-07-14

**Authors:** Kazusa Miyahara, Mizuki Hino, Zhiqian Yu, Chiaki Ono, Atsuko Nagaoka, Masataka Hatano, Risa Shishido, Hirooki Yabe, Hiroaki Tomita, Yasuto Kunii

**Affiliations:** ^1^Department of Disaster Psychiatry, International Research Institute of Disaster Science, Tohoku University, Sendai, Japan; ^2^Department of Neuropsychiatry, School of Medicine, Fukushima Medical University, Fukushima, Japan; ^3^Department of Psychiatry, Graduate School of Medicine, Tohoku University, Sendai, Miyagi, Japan; ^4^Department of Psychiatry, Tohoku University Hospital, Sendai, Miyagi, Japan

**Keywords:** schizophrenia, postmortem brain, microarray, pH, RNA integrity number

## Abstract

**Background:**

Evaluating and controlling confounders are necessary when investigating molecular pathogenesis using human postmortem brain tissue. Particularly, tissue pH and RNA integrity number (RIN) are valuable indicators for controlling confounders. However, the influences of these indicators on the expression of each gene in postmortem brain have not been fully investigated. Therefore, we aimed to assess these effects on gene expressions of human brain samples.

**Methods:**

We isolated total RNA from occipital lobes of 13 patients with schizophrenia and measured the RIN and tissue pH. Gene expression was analyzed and gene sets affected by tissue pH and RIN were identified. Moreover, we examined the functions of these genes by enrichment analysis and upstream regulator analysis.

**Results:**

We identified 2,043 genes (24.7%) whose expressions were highly correlated with pH; 3,004 genes (36.3%) whose expressions were highly correlated with RIN; and 1,293 genes (15.6%) whose expressions were highly correlated with both pH and RIN. Genes commonly affected by tissue pH and RIN were highly associated with energy production and the immune system. In addition, genes uniquely affected by tissue pH were highly associated with the cell cycle, whereas those uniquely affected by RIN were highly associated with RNA processing.

**Conclusion:**

The current study elucidated the influence of pH and RIN on gene expression profiling and identified gene sets whose expressions were affected by tissue pH or RIN. These findings would be helpful in the control of confounders for future postmortem brain studies.

## 1. Introduction

There has been increasing interest in studies on neuropsychiatric disorders conducted on the postmortem brain over the years. Schizophrenia was once called “the graveyard of neuropathologists” in 1970s, because only a few neuropathological characteristics were observed in the postmortem brain of diagnosed patients (1). However, genetic and molecular analyses have been well developed since the beginning of the 21st century (2) and postmortem brains of patients with schizophrenia were re-examined to validate genetic and molecular findings. Now, investigating the pathology of the human postmortem brain at the molecular level is critical for elucidating the pathophysiology of schizophrenia. For example, abnormal expression level of transcripts related to various biological components including γ-aminobutyric acid (GABA) signaling (3, 4), glutamatergic signaling (5), inflammatory system (6), astrocyte (7), oligodendrocyte (8), microglia (9) have been repeatedly reported and it is certain that these system are profoundly included in pathophysiology of schizophrenia. Moreover, effects of schizophrenia-associated single nucleotide polymorphisms (SNPs) on gene expression in the brain have been investigated and the increase of SNPs has been associated with aberrant gene expression and the abnormal brain structure of patients with schizophrenia (10–12). Furthermore, comprehensive analyses on gene expression have clarified the pathways that are closely associated with schizophrenia and identified the potential therapeutic agents (13, 14). There is a growing trend toward open accessibility to a large dataset of human brain such as CommonMind Consortium (15) and BrainCloud (16), lessening the hurdles for conducting postmortem brain studies. It is predicted that studies on postmortem brain would increase and would contribute to clarifying the pathophysiology of schizophrenia and other neuropsychiatric disorders.

Despite the benefits of postmortem brain studies, several inevitable issues may arise, such as heterogeneity among subjects. Various conditions other than pathophysiology of diseases, such as antemortem history, agonal state, and postmortem interval (PMI) are reflected on molecules in the postmortem brain. Therefore, it is essential to adequately evaluate and control for confounders when conducting postmortem brain studies.

Tissue pH and RNA integrity number (RIN) (17) are the most important confounders among the others, such as age of death, sex, PMI, and antemortem drugs, since these two reflects the severity of the tissue degradation (18). Tissue pH mostly depends on the agonal state (19) but not on the PMI (20). The agonal factor score, developed by Tomita et al., quantifies the influence of the agonal factors and is associated with tissue pH, demonstrating that the manner of death affects tissue pH (21, 22). Additionally, tissue pH is known to be influenced by smoking history (23) and intake of antipsychotics (24). Correspondingly, RIN has been associated with the agonal state (21) and the condition of the postmortem brain tissue after death (25). RIN and tissue pH have been known to affect each other. In previous studies, poor RNA quality was closely associated with a drop in pH (20, 26). The association between low tissue pH and RIN may be partially explained by the effect of agonal state, including hypoxia, coma, and dehydration. Severe agonal states would expose brain cells to hypoxia, causing changes of gene expression level and cell death. Hypoxia results in lower pH due to increase of lactate production (27, 28), while cell death also causes acidosis *via* release of intercellular vesicle (18). In addition, destruction of RNA along with cell damage itself may result in low tissue pH, since RNA consists of phosphoric acid. Therefore, both tissue pH and RIN reflects the severity of agonal state, which may be indicators of severity of agonal states and according changes of gene expressions. Previous studies have proven that various confounders are reflected on tissue pH and RIN. Thus, these two are essential confounders for heterogeneity adjustment.

Most postmortem brain studies include tissue pH and/or RIN as confounders; however, the effects of these two factors on gene expressions have not been fully investigated. Several studies have assessed their effects on mRNA by reverse transcription polymerase chain reaction (29–31), but the number of target genes is limited and usually under 100. A few studies (32–35) had comprehensively evaluated the effect of tissue pH on gene expression using microarray; Iwamoto et al. (33) concluded that tissue pH was associated with the altered expressions of genes involved in energy metabolism, mitochondrial function, proteolytic activities, stress-response proteins, and transcription factors, although they did not include RIN as a cofounder (34). Therefore, further studies are needed to precisely evaluate the influence of tissue pH and RIN on global gene expressions provided that control and experimental groups are examined separately.

This study aimed to identify the gene sets that were affected by tissue pH and RIN using the postmortem brain of patients with schizophrenia. We conducted microarray analysis, which was able to measure the expression levels of low-expressed genes unlike RNA-seq (36), and identified genes that were significantly correlated with tissue pH, RIN, or both. Furthermore, we assessed the function of each gene set by pathway analysis and upstream regulator analysis.

## 2. Subjects and methods

### 2.1. Human postmortem brain tissue

Postmortem brain tissue samples from patients with schizophrenia were obtained from the Fukushima Brain Bank, Department of Neuropsychiatry, School of Medicine, Fukushima Medical University, Fukushima, Japan (37). The use of postmortem human brain tissue in this study was approved by the Ethics Committee of Fukushima Medical University and Tohoku University, and complied with the principles of the Declaration of Helsinki and its later amendments. The ethics approval numbers for this study were 2021–1-127 (Tohoku University) and 1,685 (Fukushima Medical University). All procedures were performed with the written consent of close relatives. Detailed demographic information on brain tissue from the 13 subjects with schizophrenia used in this study is summarized in Table 1. The patients with schizophrenia fulfilled the diagnostic criteria established by the American Psychiatric Association (Diagnostic and Statistical Manual of Mental Disorders: DSM-IV).

**Table 1:** Demographic data of the samples.

Sample number	1	2	3	4	5	6	7	8	9	10	11	12	13
Sex	M	M	M	F	F	M	F	M	F	M	M	M	M
Age	70	75	64	87	68	60	77	66	65	74	71	39	58
PMI (hour)	31	19	6.5	5	16	46	26	6	6	20.5	17.5	35	10
Brain weight (mg)	10.1	12.5	16.1	22.3	13.8	13.7	8.4	12.3	16.2	20.2	17.4	10.8	17.6
pH	6.0	6.0	7.3	6.7	5.6	6.8	6.2	6.2	6.3	6.6	6.7	7.0	6.6
RIN	2.7	5.8	7.7	8.0	3.4	6.7	6.1	6.6	5.7	4.5	7.8	3.4	6.2

PMI, postmortem interval; RIN, RNA integrity number.

### 2.2. Tissue pH measurement of postmortem brain

The pH of postmortem brain tissue was measured as described in a previous study (38) with slight modifications. In brief, approximately 20 mg of frozen tissue was dissected and homogenized in a 5-times volume (1 ml/100 mg tissue) of nuclease-free water (Ambion, Austin, TX, USA). The pH of the homogenate was measured using Twin pH-B212 (Horiba, Kyoto, Japan).

### 2.3. RNA isolation and RNA quality measurements

Total RNA was isolated by using an AllPrep DNA/RNA/Protein Mini Kit (Qiagen, Valencia, CA, USA), and genomic DNA in the RNA samples was digested by RNase-free DNase I. The RIN was measured using the 2,100 Bioanalyzer (Agilent, Santa Clara, CA, USA) as described in a previous study (39).

### 2.4. Gene expression microarray analysis

Gene expression analysis was conducted as described in a previous study (40). In brief, biotinylated cRNA was prepared using the Ambion Illumina RNA Amplification Kit (Ambion, Austin, TX, USA). The biotinylated cRNA were hybridized to Human 6v2 Expression BeadChips (Illumina, San Diego, CA, USA). The BeadChips were then labeled with streptavidin–Cy3 (GE Healthcare, Chicago, IL, USA), washed, and scanned with Bead Station 500X (Illumina). Signal intensity (SI) of each bead was measured and normalized using average normalization by BeadStudio software Ver. 3.1 (Illumina) as previous studies (40, 41). Among the 48,702 probes, 8,286 probes had an average SI ≥ 100.

### 2.5. Statistical analysis

Correlation analysis of gene expression levels between samples was performed without applying a cutoff based on SI. For all other analyses, beads with an average SI less than 100 were excluded. Correlation analysis was performed by calculating Pearson’s correlation coefficients. For the multiple comparison tests, Benjamini–Hochberg corrections were performed. Statistical significance was defined as q value <0.05. As to gene sets which were correlated with tissue pH and RIN, we separately conducted pathway analysis using Reactome Pathway Database^1^ (42) and upstream regulator analysis using Ingenuity Pathway Analysis (IPA). We constructed a network by using Cytoscape (43) and visualized the relation between genes and reactome pathways. R programming and Excel were used for data processing.

## 3. Results

### 3.1. Influence of tissue pH and RIN on correlation of gene expression profiling

**Figure 1:**
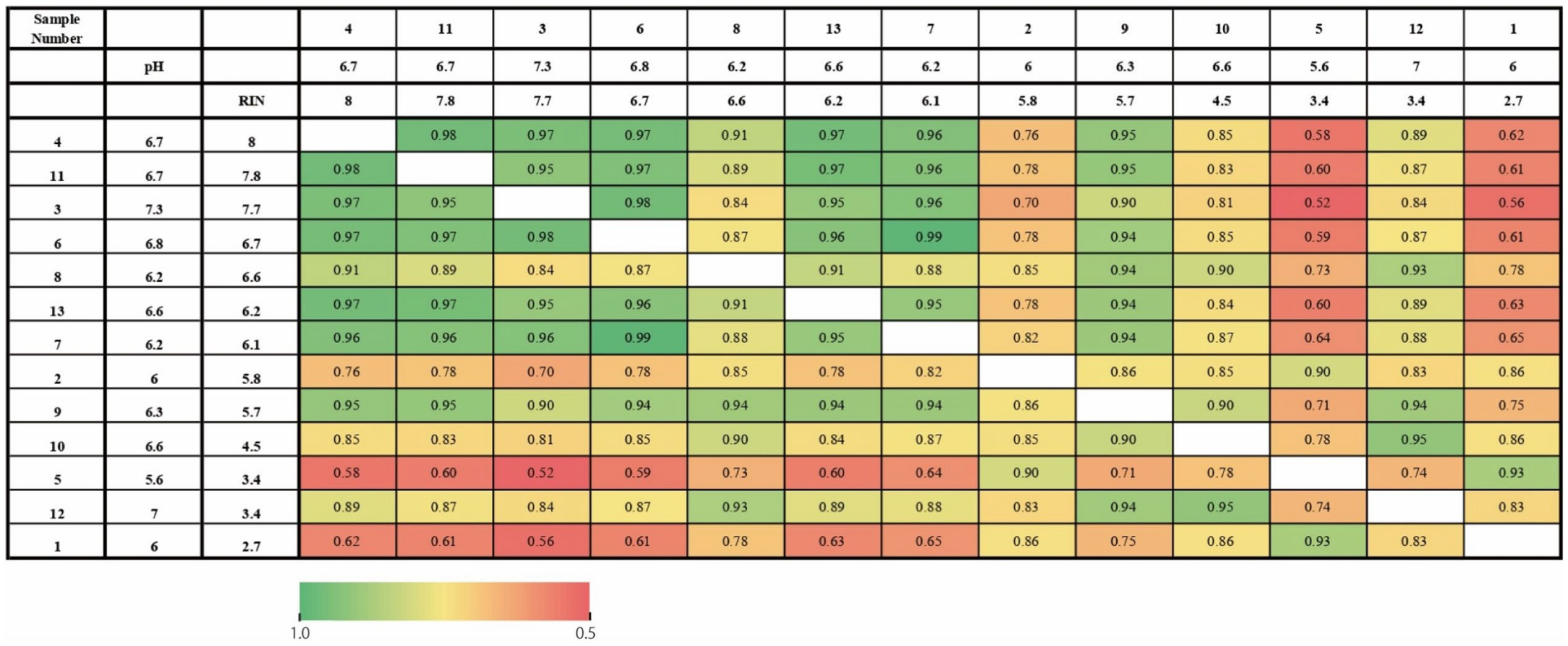
Heatmap of the correlation of gene expression profiling along with tissue pH and RIN of each subject. The numbers represent the correlation coefficients.

First, we investigated whether tissue pH and RIN similarly affected gene expression profiling on each subject. Figure 1 shows a correlation of gene expression profiling along with tissue pH and RIN of each subject. Correlation coefficients were high between the pair with similar tissue pH and RIN.

### 3.2. Correlation analysis between gene expression and covariates

**Figure 2:**
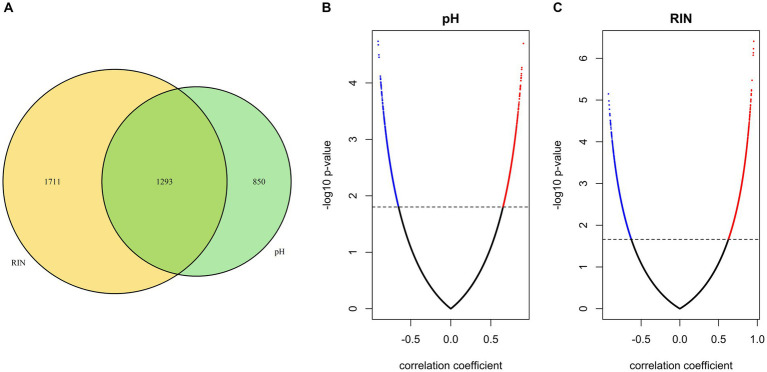
**(A)** Venn diagram of the number of genes susceptible to tissue pH, RIN, and both. **(B,C)** Volcano plots of correlation coefficient and value of *p* evaluated between gene expressions and **(B)** pH and **(C)** RIN. Significant genes are highlighted.

**Figure 3:**
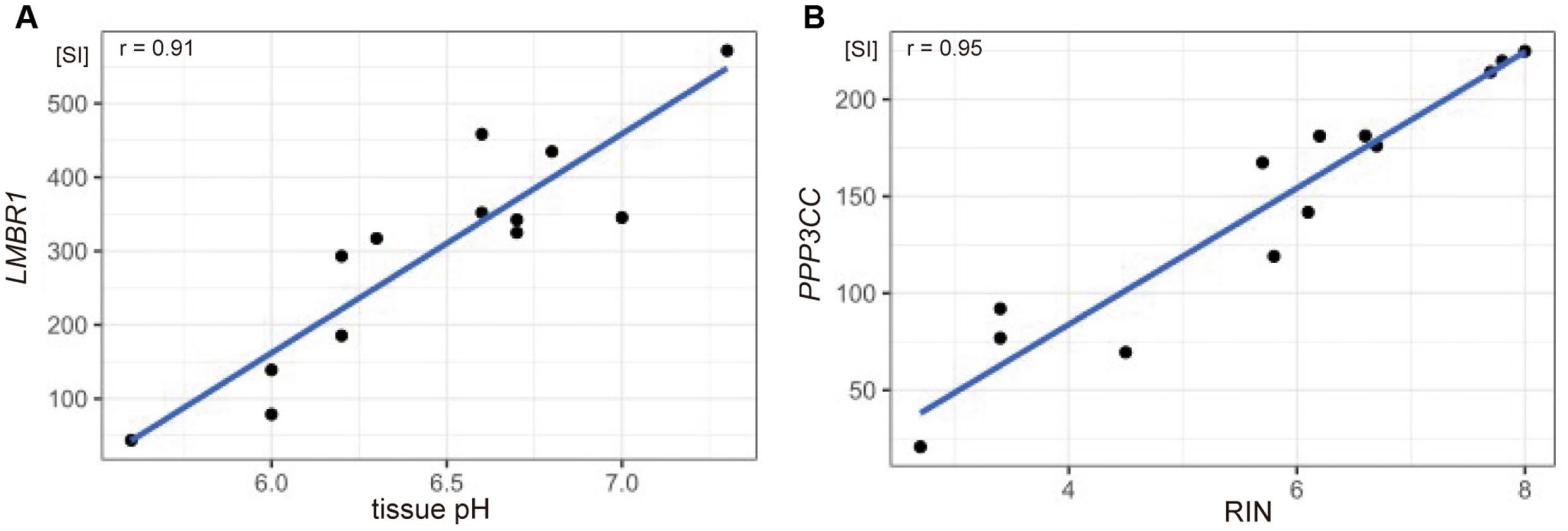
Scatter plots showing the correlation between **(A)** tissue pH and gene expression of LMBR1 (*r* = 0.91), and **(B)** RIN and PPP3CC (*r* = 0.95). SI, signal intensity.

To identify gene sets susceptible to tissue pH and RIN, we conducted correlation analysis and elucidated genes whose expression was significantly correlated with tissue pH and/or RIN (Supplementary Tables S1–S3). The expression of 2,043 genes; 3,004 genes; and 1,293 genes showed a significant correlation with tissue pH, RIN, and both, respectively (Figures 2A–C). Conversely, 4,532 genes were not significantly correlated with pH or RIN. Among the genes susceptible to tissue pH and RIN, *LMBR1* and *PPP3CC* were most highly correlated with tissue pH and RIN, respectively (Figure 3A: *LMBR1*; *r* = 0.91, Figure 3B: *PPP3CC*; *r* = 0.95).

### 3.3. Pathway analysis on covariates-susceptible genes

**Figure 4:**
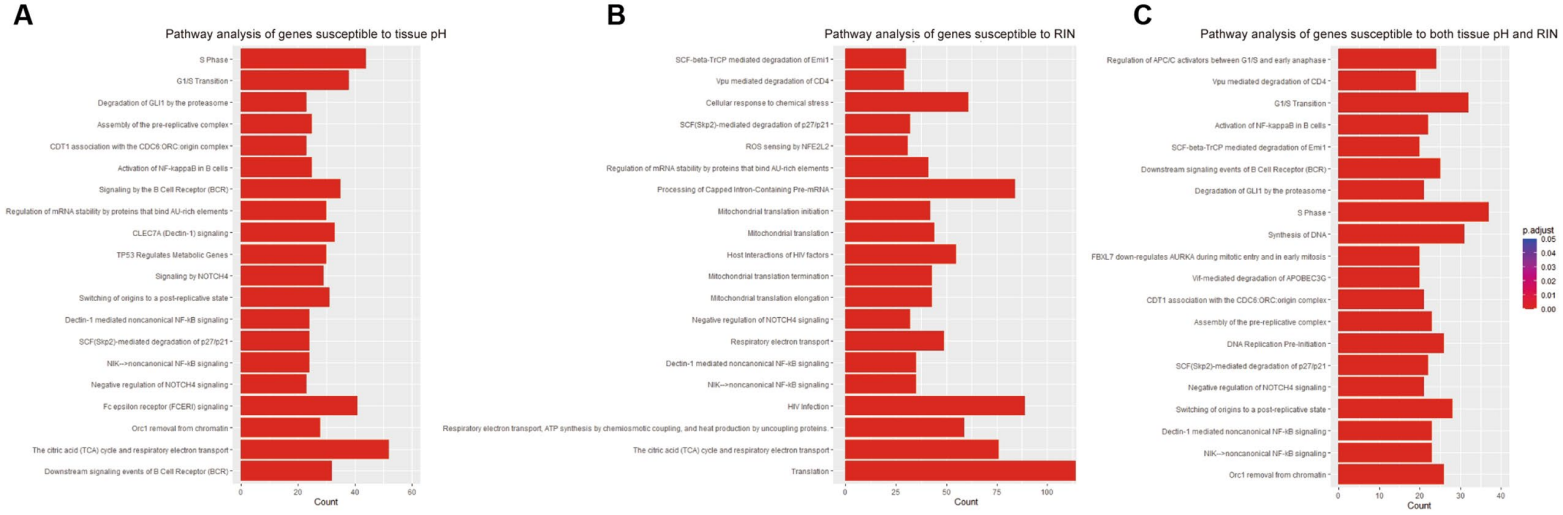
Results of pathway analyses of the gene sets susceptible to **(A)** tissue pH, **(B)** RIN, and **(C)** both.

**Figure 5:**
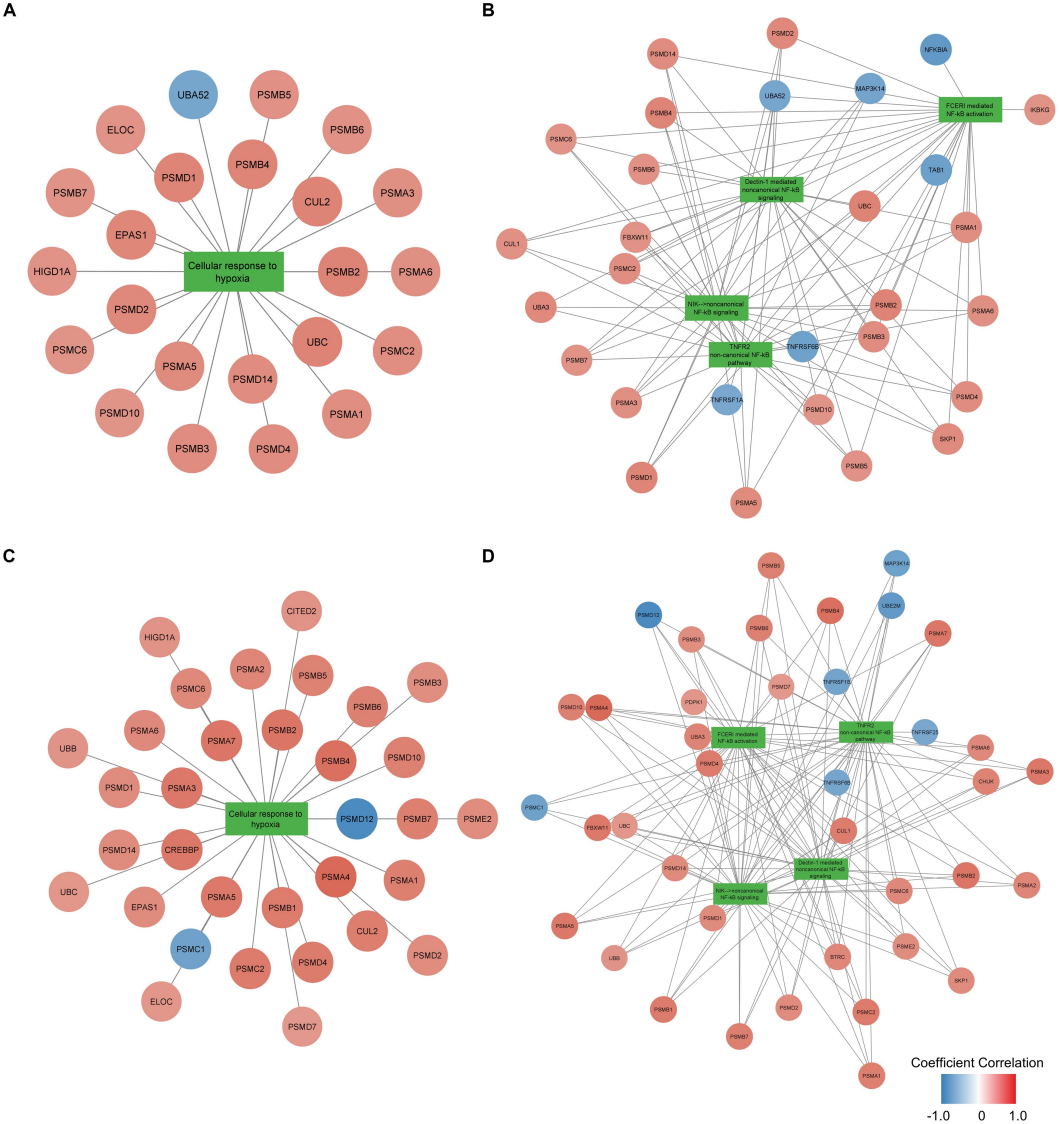
Network of genes significantly correlated with pH belonging to the **(A)** hypoxia-or **(B)** NF-kappaB-related pathway and network of genes significantly correlated with RIN belonging to the **(C)** hypoxia-or **(D)** NF-kappaB-related pathway. Node color indicates the coefficient correlation. Node size indicates the significance of the correlation; larger sizes indicate smaller *p-*values.

To examine the function of identified gene sets susceptible to tissue pH and RIN, we carried out pathway analysis using Reactome Pathway Database. Genes susceptible to tissue pH were significantly enriched in pathways that were related to energy production, immune system, and cell cycle (Figure 4A). Genes susceptible to RIN were significantly enriched in pathways that were related to energy production, immune system, and RNA processing (Figure 4B). Genes susceptible to both tissue pH and RIN were significantly enriched in pathways that were related to energy production and immune system, including nuclear factor kappa B (NF-kappaB) signaling (Figure 4C). Supplementary Figures S1, S2 show concept networks of the gene set susceptible to tissue pH and RIN, which reflect correlation coefficient of each gene with top 6 pathways. The full results of enrichment analysis are shown in Supplementary Tables S4–S6. As a result of upstream regulator analysis, hypoxia inducible factors (HIF) 1a was found to be a significant regulator of genes susceptible to tissue pH (*p* = 9.1 × 10^−4^), although regulation of its downstream molecules was varied (Supplementary Table S7). Significantly-correlated genes belonging to the hypoxia-or NF-kappaB-related pathway are visualized as a network with coefficient correlation and value of *p* (pH: Figures 5A,B; RIN: Figures 5C,D). Genes belonging to pathway related to HIFs included those coding proteins associated with ubiquitin–proteasome pathway, such as proteasome 26S subunit ubiquitin receptor, non-ATPase 4 (PSMD4) and proteasome 20S subunit beta 6 (PSMB6). On the other hand, genes belonging to pathway related to NF-kappaB include those coding tumor necrosis factor receptor superfamily (TNFRSF), such as TNFRSF1B and TNFRSF6B.

## 4. Discussion

In this study, we used the postmortem brains of 13 patients with schizophrenia and examined the influence of tissue pH and RIN on global gene expression profiling. We identified 2,043 genes (24.7%) susceptible to tissue pH; 3,004 genes (36.3%) susceptible to RIN; and 1,293 genes (15.6%) susceptible to both, and the remaining genes were not susceptible to pH or RIN. Additionally, we conducted pathway analysis and clarified that genes susceptible to pH were enriched in pathways that were related to energy production, immune system, and cell cycle, whereas genes susceptible to RIN were significantly enriched in pathways that were related to energy production, immune system, and RNA processing. To the best of our knowledge, this study is the first to report on these findings and would have an impact on the future postmortem studies for controlling confounders.

The characteristics of the gene sets susceptible to tissue pH and RIN had commonalities. Both gene sets were enriched in pathways associated with energy production and immune system. This may be attributed to the hypoxia exposure during the agonal state affecting tissue pH, RIN, and specific gene expression profiling. Hypoxia exposure is known to induce abnormal energy production and immune system functioning (44, 45).

Hypoxia-dependent change of energy production is mainly due to HIFs, a transcription factor activated during conditions of hypoxia (46). Although HIFs are usually hydroxylated by prolyl hydroxylases (PHD) and degradated by ubiquitin-proteasome system, HIFs are activated in hypoxic conditions because exposure decreases PHD enzymatic activity. HIFs are known to impact energy metabolism. For example, HIFs upregulates lactate dehydrogenase and downregulates pyruvate dehydrogenase, resulting in decreased energy production in the tricarboxylic acid cycle and electron transport system (44). Because tissue pH and RIN were significantly correlated with gene expression related to energy production, it is suggested that these confounders have reflected metabolic change during the agonal state. Since variable proteasome proteins were positively correlated with tissue pH and RIN as shown in Figures 5A,C, activation of proteasome may be crucial for adaptation to hypoxia, which is consistent with previous studies reporting that PSMD4 and PSMB6 are activated in hypoxia environment (47, 48). In addition to regulation of metabolic enzyme due to HIFs, miRNAs located downstream of HIFs (called hypoxamiRs) are also acting in adaptation to hypoxia (49, 50). hypoxamiRs are small non-coding RNAs that regulate gene expression by forming complexes and degrading mRNAs with complementary sequences. Such hypoxamiRNAs effect on metabolic and immune pathways by downregulation of specific mRNA (51, 52). The above adaptive responses to hypoxia exposure occur when downstream genes of HIFs, including hypoximiRNAs, alter their gene expression patterns. *In vivo* experiments have shown that HIF1 and HIF2 play a role in the production of energy sufficient for survival in hypoxic environments (53) and in recovery from hypoxic exposure in the brain (54).

Meanwhile, the link between schizophrenia and aberrant energy production has been repeatedly reported (55, 56). In previous postmortem brain studies, the hypoxia-related pathway was activated and mitochondrial dysfunction was seen in the postmortem brains of patients with schizophrenia, despite controlling multiple confounders, including tissue pH and RIN (57, 58). Thus, it is likely that schizophrenia, to some extent, impacts the metabolism process in the human brain. Nevertheless, tissue pH and RIN may be critical markers for correcting the agonal metabolic change to precisely distinguish pathophysiology of schizophrenia and the artifact of the postmortem brain study.

Hypoxia exposure also induces immune response. Inhibition of PHD activates NF-kappaB, an essential transcription factor for inducing immune response (45). Since HIFs and NF-kappaB promote expression of the each other (59), hypoxia response and immune response are considered closely related (60). Among the genes belonging to pathways of NF-kappaB, TNFRSF were negatively correlated with pH and RIN, as shown in Figures 5B,D. TNFRSF has been known to include essential molecules for immune activity (61), and the current results suggest TNFRSF also plays a crucial role for reaction to hypoxia. NF-kappaB is a molecule often associated with schizophrenia. NF-kappaB was observed to be over activated while its inhibitor, human immunodeficiency virus enhancer binding protein 2 (HIVEP2), was inactivated in the postmortem brain of patients with schizophrenia (62, 63). Additionally, Murphy CE et al. reported that low tissue pH was associated with the high expression of NF-kappaB in patients with schizophrenia (64), which is consistent with our findings that tissue pH affects genes related to the immune system including NF-kappaB signaling. Therefore, activation of NF-kappaB is important for the pathophysiology of schizophrenia and its expression level should be controlled for tissue pH and RIN.

The pathways unique to pH-susceptible genes were related to cell cycle. Investigations on cells outside the brain have shown that hypoxia exposure affects cell cycle transition (65–67). For example, NF-kappa B pathway activated by hypoxia exposure promotes cell cycle to enhance the cell proliferation in pulmonary arterial smooth muscle cells. It is likely that similar cell cycle promotion had occurred during the agonal state as a response to hypoxia exposure. Conversely, RNA processing was the pathway unique to RIN-susceptible genes. This may be attributed to the disruption of RNA dynamics during the agonal state due to the reduction of RIN induced abnormal expression of the gene set related to RNA processing.

Our finding, that nearly 5,000 genes were affected by tissue pH or RIN, underlines the importance of controlling the confounders. These gene sets include genes commonly used as reference genes for postmortem brain studies (68), such as *ACTB, B2M, POLR2A, and RPLP0CTB.* In these for genes, expression levels of *ACTB* and *RPLP0* were correlated with both tissue pH (*ACTB*: *r* = 0.75; *RPLP0*: *r* = −0.69) and RIN (*ACTB*: *r* = −0.90; *RPLP0*: *r* = 0.70). Expression level of *POLR2A* were correlated with only tissue pH (*r* = −0.68), while that of *B2M* were correlated with only RIN (*r* = 0.72). On the other hand, other reference genes like *GAPDH* and *TFRC* were not included. These results suggest that some reference genes were susceptible to tissue pH, RIN or both. Therefore, when conducting normalization of gene expression level using reference genes, it should be noted that reference genes may be affected by agonal states. In addition, the current identified gene sets include genes that have been linked to schizophrenia. For example, we demonstrated that GAD, protein phosphatase 3 catalytic subunit gamma (PPP3CC) and glutamate ionotropic receptor NMDA type subunit (GRIN) were susceptible to tissue pH and RIN, all of which were associated with schizophrenia in previous postmortem brain studies (3, 69, 70). When examining the expression level of these genes in postmortem brain, it is essential to control tissue pH and RIN as confounders. Cyclic AMP-responsive element binding protein (CREBBP) was identified as RIN-susceptible gene; however, its expression level has not been investigated while controlling RIN as a continuous confounder (71, 72). Therefore, the current insight that CREBBP is upregulated in patients with schizophrenia (73) may not be a solid theory and further studies are required.

Moreover, our findings may be the key for integrating inconsistent insights about specific gene expression level. For example, synaptophysin (SYP), an essential molecule for neurotransmission, has been reported to be reduced in patients with schizophrenia (74–76); however, a contradicting report (77) did not include tissue pH as a confounder. Because this study demonstrated that SYP was susceptible to tissue pH, considering adequate confounders may consolidate ambiguous findings.

The gene set identified susceptible to tissue pH in this study may reflect not only agonal state but also pathophysiological change due to schizophrenia. Although we considered tissue pH and RIN as confounders, several previous studies have reported that low pH may be a part of pathophysiology of psychosis (66, 78, 79). A study (78) conducted a meta-analysis evaluating tissue pH of postmortem brain from patients with schizophrenia and bipolar disorder and demonstrated that tissue pH of the patients was significantly lower than that of controls even when several confounders such as PMI and antemortem intake of antipsychotics were considered. Lower tissue pH in brain of patients with schizophrenia has been associated with aberrant metabolism and hypoxia-related changes in gene expression (58), which are overlapped with the transcriptome profile associated with pH and RIN in our current study. Therefore, caution is needed to interpret gene expression data from the postmortem brain, especially when the genes associated with pH and RIN would be detected as differentially expressed between tissues from control and patients with schizophrenia.

Our study has several limitations. First, effects of other confounders such as PMI and tissue processing could not be excluded. Second, we were unable to investigate the effects of tissue pH and RIN on all transcripts. Future studies are needed to fully investigate the effect of tissue pH and RIN using RNA-seq. Third, we evaluated gene expression profile in a single region. Influence of tissue pH and RIN in multiple regions remains unknown. Furthermore, as we used postmortem brain of patients with schizophrenia, there might be a potential effect of pathophysiology of the disease. Thus, further investigations are required to confirm our findings.

It should be noted that the exact magnitude of effect was not examined for individual genes. Ideally, it would be necessary to measure individual genes expression levels accurately by quantitative polymerase chain reaction and quantify protein expression levels by immunohistochemistry or western blot. Such additional experiments would clarify the details of the changes in gene expression patterns and are necessary for future postmortem brain research.

In conclusion, our study provides new insight into the effect of tissue pH and RIN on gene expression profile in postmortem brain. Our findings indicate that tissue pH and RIN oscillate the expression level of specific gene sets and that these two are critical confounders in postmortem brain analysis. These findings promote the need to adequately control confounders, subsequently paving the way to upgraded postmortem brain studies for elucidating pathophysiology of schizophrenia.

## Data availability statement

The data presented in the study are deposited in the Gene Expression Omnibus database, accession number GSE235055 and are available at the following URL: https://www.ncbi.nlm.nih.gov/geo/query/acc.cgi?acc=GSE235055.

## Ethics statement

The studies involving human participants were reviewed and approved by ethics committees of Fukushima Medical University and the Tohoku University Graduate School of Medicine. The patients/participants provided their written informed consent to participate in this study.

## Author contributions

KM, MHa, MHi, CO, ZY, HT, and YK designed the study. MHi, ZY, and YK performed the experiments. MHa, MHi, RS, AN, HY, HT, and YK collected postmortem brain samples and clinical information. KM, MHi, ZY, and YK undertook the statistical analysis. KM wrote the first draft. All authors contributed to and have approved the final manuscript.

## Funding

This work was supported by Japan Agency for Medical Research and Development (AMED) – JP22dm0207074 (YK) and AMED – JP22wm0425019 (HY); the Grant-in-Aid for Scientific Research on Innovative Areas from the Ministry of Education, Culture, Sports, Science, and Technology of Japan under Grant Number JP221S0003, JP24116007 (HT), and JP21H00180 (YK), the Promotion of Science Grant-in-Aid for Scientific Research on Priority Areas (20023005); and the Collaborative Research Project of Brain Research Institute, Niigata University under Grant Number 2017–2806 (HT), and 22002 (YK).

## Conflict of interest

The authors declare that the research was conducted in the absence of any commercial or financial relationships that could be construed as a potential conflict of interest.

## Publisher’s note

All claims expressed in this article are solely those of the authors and do not necessarily represent those of their affiliated organizations, or those of the publisher, the editors and the reviewers. Any product that may be evaluated in this article, or claim that may be made by its manufacturer, is not guaranteed or endorsed by the publisher.

## Footnotes

^1^ https://reactome.org/

## Abbreviations

CREBBP: cyclic AMP-responsive element binding protein
GAD: glutamic acid decarboxylase
HIF: hypoxia inducible factors
IPA: ingenuity Pathway Analysis
PMI: postmortem interval
PSMB6: proteasome 20S subunit beta 6
PSMD4: proteasome 26S subunit ubiquitin receptor, non-ATPase 4
RIN: RNA integrity number
SI: signal intensity
TNFRSF: tumor necrosis factor receptor superfamily
