# Supplementary material for: The influence of tissue pH and RNA integrity number on gene expression of human postmortem brain
Source: Front Psychiatry. 2023 Jul 14;14:1156524. doi: 10.3389/fpsyt.2023.1156524 (PMC10379646; doi:10.3389/fpsyt.2023.1156524)
Supplement: Supplementary file 2 [file Data_Sheet_2.DOCX]

Supplementary Material

The influence of tissue pH and RNA integrity number on gene expression of human postmortem brain

Kazusa Miyahara, Masataka Hatano, M.D., Mizuki Hino, Ph.D., Risa Shishido, M.D., Atsuko Nagaoka, M.D., Ph.D., Chiaki Ono, Ph.D., Zhiqian Yu , Ph.D., Hirooki Yabe, M.D., Ph.D., Hiroaki Tomita, M.D., Ph.D. and Yasuto Kunii M.D., Ph.D.*

*** Correspondence:** Yasuto Kunii: kunii@med.tohoku.ac.jp

# Supplementary Figures


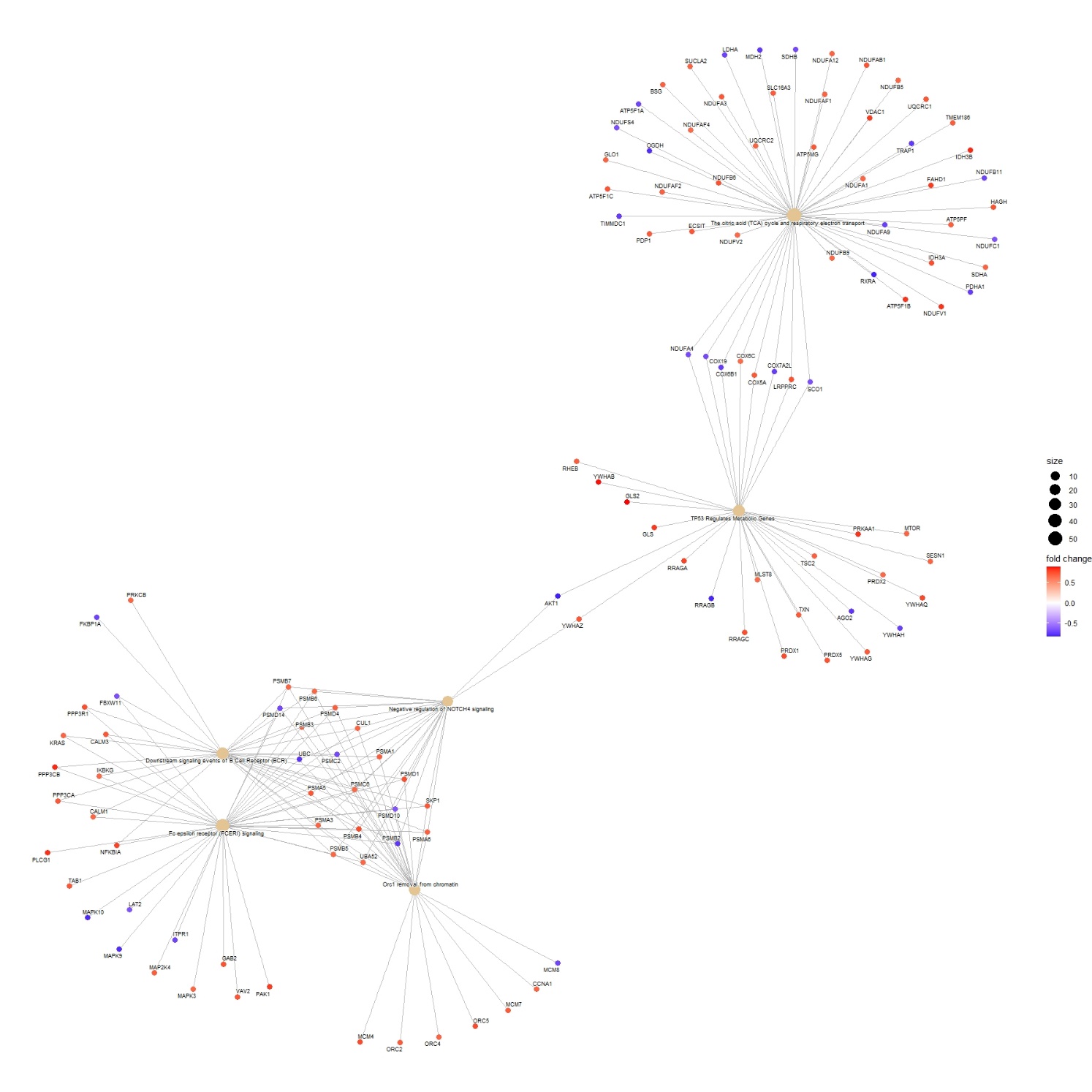


**Supplementary Figure 1.** A concept network of gene sets susceptible to tissue pH which reflects correlation coefficient of each gene with top 6 pathways. A color of the node represents the correlation coefficient.


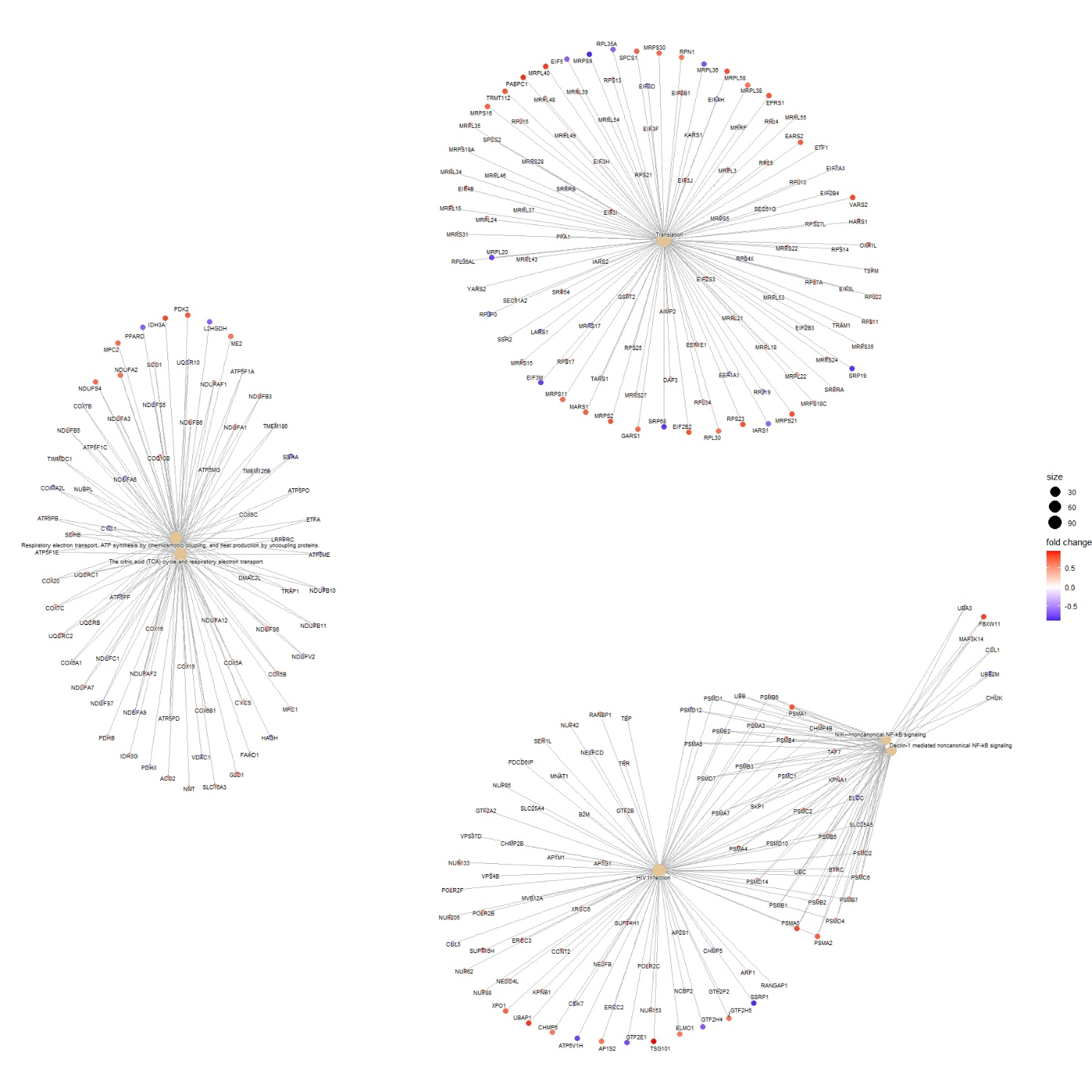


**Supplementary Figure 2.** A concept network of gene sets susceptible to tissue pH which reflects correlation coefficient of each gene with top 6 pathways. A color of the node represents the correlation coefficient.
